# Judging the position of the artificial hand induces a “visual” drift towards the real one during the rubber hand illusion

**DOI:** 10.1038/s41598-018-20551-6

**Published:** 2018-02-07

**Authors:** Roberto Erro, Angela Marotta, Michele Tinazzi, Elena Frera, Mirta Fiorio

**Affiliations:** 1 Department of Neurosciences, Biomedicine and Movement Sciences, Università di Verona, Verona, Italy; 2 Center for Neurodegenerative Diseases (CEMAND), Department of Medicine, Surgery and Dentistry “Scuola Medica Salernitana”, University of Salerno, Baronissi (SA), Italy; 3 Neurology Unit, Neuroscience Department, AOUI, Verona, Italy

## Abstract

When subjects look at a rubber hand being brush-stroked synchronously with their own hidden hand, they might feel a sense of ownership over the rubber hand. The perceived mislocalization of the own hand towards the rubber hand (proprioceptive drift) would reflect an implicit marker of this illusion occurring through the dominance of vision over proprioception. This account, however, contrasts with principles of multisensory integration whereby percepts result from a “statistical sum” of different sensory afferents. In this case, the most-known proprioceptive drift should be mirrored by complementary *visual* drift of the rubber hand in the opposite direction. We investigated this issue by designing two experiments in which subjects were not only requested to localize their own hand but also the rubber hand and further explored the subjective feeling of the illusion. In both experiments, we demonstrated a (visual) drift in the opposite direction of the proprioceptive drift, suggesting that both hands converge toward each other. This might suggest that the spatial representations of the two hands are integrated in a common percept placed in between them, contradicting previous accounts of substitution of the real hand by the rubber hand.

## Introduction

The sense of body ownership refers to the implicit and explicit experience that your body belongs to you and constitutes one of the fundamental basic aspects of self-awareness^1^. Several lines of evidence suggest that the sense of body ownership it is malleable. In fact, both embodiment of extra-corporeal objects and disownership of own body parts have been reported [for a review see^2^]. One way to experimentally manipulate the sense of body ownership is the rubber hand illusion (RHI)^3^.

In the RHI simultaneous brush stroking of a subject’s hidden hand and a visible rubber hand induces a transient illusion of the latter to “feel like it’s my hand”^3^. In other words, matching visual and tactile information gives rise to a sense of ownership over the rubber hand. As a result of the illusion, the location of the subject’s hidden hand is perceived as closer to the viewed location of the rubber one and, hence, the RHI can be implicitly measured as the localization bias (the so-called *proprioceptive drift*) of the perceived position of the subject’s own hand towards the rubber hand^3,4^.

Beyond the synchronous visual-tactile stimulation, other levels of intermodal congruence are needed for the illusion to occur. In fact, integrating information across several sensory systems poses the brain a computationally complex problem. For instance, it has been shown that orienting the rubber hand at 90 or 180 degrees with respect to the subject’s own hand abolishes the illusion, suggesting that visual-proprioceptive congruence is also necessary^4^. Such evidence implies, from a Bayesian perspective of multisensory integration^5^, that the illusion could only occur if the weighting of conflicting sensory information and their subsequent integration result in a *statistically plausible* compromise. On the contrary, whenever a gross mismatch is introduced, the illusion would vanish^4^.

Several studies have demonstrated that percepts resulting from the integration of different sensory inputs dynamically depend on the relative reliability of each sensory modality. For instance, when people look at an object while concomitantly exploring it with their hands, vision often “dominates” the integrated visual-haptic percept^6–8^. Conversely, when vision is experimentally blurred, the resulting percept is mostly driven by haptic afferents^9,10^. A general principle for multisensory integration has been hence proposed that minimizes variance in the final estimate, suggesting that it results from the “statistical sum” of different sensory information, each of which is weighted according to its reliability^11^. Statistically optimal integration has been demonstrated for different sensory modalities, including vision and proprioception^12^.

Previous accounts of the RHI postulated a three-way weighted integration between vision, touch, and proprioception: vision of the tactile stimulation on the rubber hand captures the tactile sensation on the subject’s own hand, and this visual capture results in a mislocalization of the felt position of the own hand towards the location of the visual percept^13^. In other words, provided that intermodal congruence is present, vision “dominates” over proprioception^14,15^ and results in the proprioceptive drift. This account, however, contrasts with the aforementioned principles of multisensory integration and would rather support the idea that the integrated percept is based on vision alone, through a “winner-takes-all” mechanism^16^. However, several studies of multisensory integration would argue against the latter mechanism, showing that bimodal stimuli evoke integrated responses that are quantitatively different from those evoked by their unimodal components separately^17–20^.

Following such a rationale, Fuchs *et al*. recently designed an experiment in which subjects were requested to localize not only the position of their own hand (as in the classic RHI paradigm), but also of the rubber hand^21^. They argued that there exists an opposite perceptual drift of the rubber hand towards the participants’ own hand, suggesting that visual and proprioceptive information are indeed “fused” into an intermediate percept^21^. However, the results of that study did not clearly demonstrate an actual drift of the rubber hand towards the participants’ hand,^21^. Although after stroking the “visual” percept shifted laterally towards the veridical position of both hands (i.e., closer to both hands as there was less “undershooting” towards the midline), it was still placed between the midline and the veridical position of the rubber hand and not between the two hands. Such a finding was interpreted as a *relative* approach of the rubber hand to the subjects’ hand, although it could simply suggest an *absolute* approach of the perceived location of the rubber hand towards its veridical position. In other words, subjects were more precise in localizing the rubber hand after the stroking.

Several reasons might account for the fact that they only succeeded in demonstrating a *relative* drift of the rubber hand towards the subjects’ hand, including the “midline bias” (i.e., the localization bias towards the midline prior to the stroking and thus independent of the illusion) and the short time of stroking (i.e. 60 sec in the first trial and 30 sec thereafter)^21^.

Therefore, to confirm that there is a reciprocal attraction of the two hands during the RHI and overcome the aforementioned potential issues, we designed two experiments. To minimize the so-called “midline bias”^12,21,22^ we interchanged the position of the two hands. To increase the magnitude of the drift(s), we extended the duration of the stroking to 2 minutes. Moreover, differently from Fuchs *et al*.^21^, our participants did not use pointing movements with the contralateral arm to localize either hand, but were asked to verbally report the location of either hand on a ruler as detailed below.

In both experiments and in line with Fuchs *et al*.^21^, we expected an actual approach of either hand towards each other, regardless of the lateral distance of the subject’s own hand to the midline.

We also aimed to explore the subjective correlates of the illusion, by administering the classic questionnaire of the RHI^3^.

## Methods

### Participants

As described in detail below, we performed two different experiments. For each, 16 healthy subjects were recruited. There was no difference in terms of age (experiment1: 23.2 ± 2.9 vs experiment 2: 23.8 ± 3.6, p > 0.05) or gender distribution (experiment1: 9 male vs experiment 2: 7 male, χ^2^ = 0.5, df = 1, p > 0.05) between the two groups. For each group, all participants but one were right-handed. The study was approved by the local ethical committee of the University of Verona and was conducted in accordance with the approved guidelines. All subjects were informed about the nature of the project and signed a written consent form prior to the testing.

### Experimental set-up

**Figure 1:**
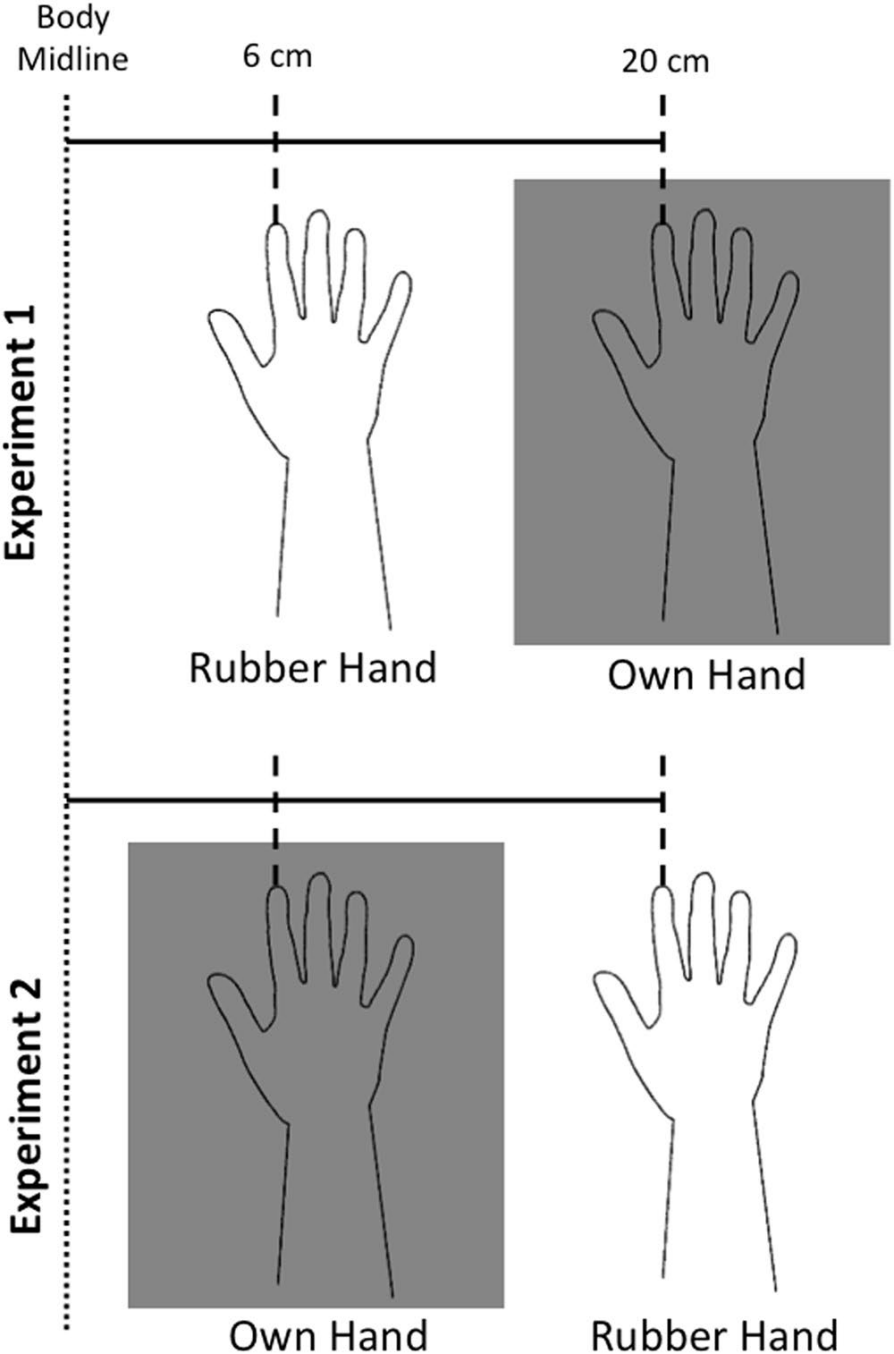
Set-up in the two experiments. This schematic representation shows that the position of the own and rubber hand was inter-changed in the two experiments. The grey square indicates that the participant’s own hand was covered by a black board during the stroking. This implies that the source of visual information (i.e., the rubber hand) was kept constant, although the “focus” was switched from one hand to the other across the two target conditions (see text for details).

We performed two different experiments: the only difference between the two experiments was the relative position of the rubber hand to the subject’s own hand (Fig. 1). Hence, the experimental set-up will be described once for both experiments. In experiment 1, a unisex cosmetic reproduction of a right hand was placed on a table, the tip of the index finger being 6 cm lateral to the participant’s body midline and 14 cm medial to the participant’s own hand index (Fig. 1, upper panel). In experiment 2, the position of the two hands was inverted: the participant’s right hand index was placed 14 cm medial to the rubber hand and 6 cm lateral to the midline (Fig. 1, lower panel).

In both experiments, only the rubber hand was visible during the stroking, whereas the participant’s own hand was covered with a black board. Subjects kept their left hand, palm facing down, on their left thigh. A black cloth anchored to the participants’ neck and shoulders on the one side and to the set-up on the other, covered the entire upper-body to neutralize any visual feedback.

### Procedure

Only the right hand was tested. As previously described^3,4,13,23^, subjects were asked to look at the rubber hand, while two paintbrushes were used to stroke both the rubber hand and the participant’s hidden hand either synchronously or asynchronously for 2 min, in separated sessions, counterbalanced across the subjects. Stroking was applied on the tip of the index finger in a rhythmic manner at about 1 Hz.

Synchronous brush-stroking of the real and the artificial hand is known to induce the RHI;^3,4,13,23,24^ asynchronous stroking is commonly used as a control procedure as it does not generally evoke the illusion^3^.

For both experiments, two *target* conditions were introduced^21^. In the “target-OWN” condition, before and after each stroking session, participants had to verbally refer the perceived position of their own index finger on a ruler positioned in front of them, just above a black board covering both hands. The ruler onset and offset numbers changed every time to avoid response biases. In the “target-RUBBER” condition, subjects were instead asked to localize the position of the rubber hand index on the ruler. During the localization judgments (in both target conditions), participants were instructed not to move their own hand. For the “target-RUBBER” condition, baseline localization of the rubber hand (i.e. prior to the stoking) was obtained after subjects had looked at it for 2 min without any stroking.

Since the current experiments were nested in a bigger project on the RHI, subjects performed the “target-OWN” condition always before the “target-RUBBER” condition. Subjects were tested for the two *target* conditions at least four weeks apart.

### Measures of the illusion

We calculated the difference between the perceived location of the subject’s own hand (in the “target-OWN” condition) and of the rubber hand (in the “target-RUBBER” condition) from their veridical positions. This difference was called displacement error and was computed twice: before and after stroking. As an implicit measure of the illusion^3,4,13,23^, we calculated the difference (called drift) between the displacement errors before and after the stroking in both target conditions, with positive values indicating an approach to the other hand in both experiments. This difference hence reflects the proprioceptive and visual drifts in the “target-OWN” and “target-RUBBER” conditions, respectively. Compared to pointing movements, the ruler has the advantage of excluding any potential bias due to mere movement execution. Namely, in the pointing movements method, the proprioceptive judgment could entail errors intrinsically linked to both the localization of the contralateral arm and its movement to the target. For this reason, we wanted to overcome this potential bias by asking our participants to verbally report with their eyes open the localization of either (hidden) hand on a ruler. Several groups including ours have adopted this approach with consistent results^4,23^.

Subsequent to the localization judgment after the tactile stimulation (as described above), participants were asked to rate on a numerical rating scale (from −3 = completely disagree to + 3 = completely agree, and an additional anchor in the middle indicating a neutral response) the degree of agreement or disagreement with nine sentences assessing the subjective experience they had during the stroking (Table 1; adapted from^3^). The sentences were read aloud by the experimenter in a randomized order of presentation across participants^23^ and they had to write their ratings on a paper template. Only whole numbers were hatch marked on the template at every 1 cm, but subjects were instructed that they could indicate any points on the scale (measures were calculated in cm and rounded up to one decimal). As to the statements, the first three are thought to directly correlate with the presence of the illusion^3^, addressing illusory touch and feeling of ownership. In detail, Statement 1 (S1) refers to an illusory localization of touch (“It seemed as if I were feeling the touch of the paintbrushes in the location where I saw the rubber hand touched”), S2 refers to a causal link between vision and touch (“It seemed as though the touch I felt was caused by the paintbrushes touching the rubber hand”), and S3 more directly refers to the illusory feeling of ownership (“I felt as if the rubber hand was my own hand”). Conversely, the other six sentences have been originally developed as “control” statements^1^ (S4: “It felt as if my hand were drifting toward the rubber hand”; S5: “It seemed as if I might have more than one hand or arm”; S6: “It seemed as if the touch I was feeling came from somewhere between my own hand and the rubber hand”; S7: “It felt as if my hand were turning ‘rubbery’”; S8: “It appeared as if the rubber hand were drifting towards my hand“; S9: “The rubber hand began to resemble my own hand”).

**Table 1:** The nine statements included in the questionnaire to explore the subjective feelings of the illusion.

S1 - “It seemed as if I were feeling the touch of the paintbrushes in the location where I saw the rubber hand touched”
S2 - “It seemed as though the touch I felt was caused by the paintbrushes touching the rubber hand”
S3 - “I felt as if the rubber hand was my own hand”
S4 - “It felt as if my hand were drifting toward the rubber hand”
S5 - “It seemed as if I might have more than one hand or arm”
S6 - “It seemed as if the touch I was feeling came from somewhere between my own hand and the rubber hand”
S7 - “It felt as if my hand were turning *rubbery*”
S8 - “It appeared as if the rubber hand were drifting towards my hand”
S9 - “The rubber hand began to resemble my own hand”

Subsequently, the experimenter placed the participants’ hand under the black board as described above, while they were instructed to keep their eyes closed.

Finally, in order to test whether subjects were accurate in localizing the own and the rubber hand before stroking, we averaged the displacement errors obtained in the two conditions (synchronous and asynchronous) prior to stroking and called this value *displacement_pre*.

### Statistical analysis

Given that data were not normally distributed (Shapiro-Willks, p < 0.050), non-parametric analyses were performed. Thus, for each variable of interest (i.e. drift, displacement_pre, questionnaire scores) separate Friedman tests were performed for each experiment. When the model yielded significant results, post-hoc test were performed by means of the Wilcoxon signed-rank test. Finally, Mann–Whitney U test was used to assess differences between the two experiments. p < 0.05 was deemed significant. Possible correlations between the drifts and the agreement with the statements were explored with the Spearman’s test. All analyses were performed using STATA v.11 (STATACorp, USA).

## Results

### Experiment 1

**Figure 2:**
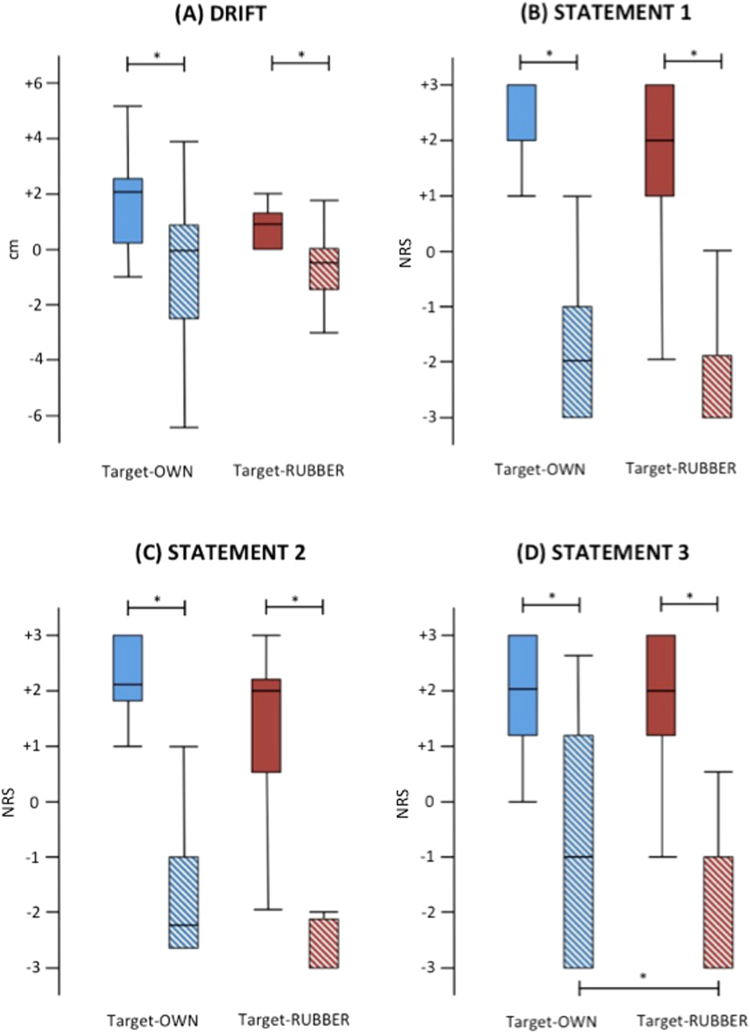
Boxplot of the drifts and statements 1 to 3 in experiment 1. The typical pattern of the rubber hand illusion in the synchronous (plain columns) compared to the asynchronous condition (striped columns) was observed for both target conditions. Moreover, a significant difference as to statement 3 was observed when comparing the two target conditions after asynchronous stroking. Asterisks indicate significant comparisons (p < 0.050).

Friedman test yielded significant results for the drift (χ^2^ = 14.76, p = 0.004). Post-hoc tests (Bonferroni-corrected critical p ≤ 0.025) demonstrated this was because of higher scores after the synchronous than asynchronous stroking (Fig. 2A), in both target-OWN (z = 2.23, p = 0.007 and target-RUBBER condition (z = 2.89, p = 0.004). No differences were seen between the two target conditions.

Similarly, the statements S1 to S3 were found to be significant (Friedman test, S1: χ^2^ = 32.31, S2: χ^2^ = 29.94, S3: χ^2^ = 25.44, all p < 0.001). This was because of higher scores after the synchronous than asynchronous stroking (i.e., more agreement; Fig. 2B–D), in both the target-OWN (S1: z = 3.50, p < 0.001; S2: z = 3.49, p = 0.003; and S3: z = 2.93, p < 0.01) and the target-RUBBER condition (S1: z = 3.50, p < 0.001; S2: z = 3.45, p = 0.004; and S3: z = 3.53, p < 0.001). There were no differences between the two target conditions, with exception of S3 where after the asynchronous stroking lower scores (i.e., more disagreement) were observed in the target-RUBBER condition as compared to the target-OWN condition (Fig. 2D, z = 2.47, p = 0.013).

As to the other statements, only S7 reached statistical significance (Friedman test, χ^2^ = 8.47, p = 0.037). This owed to lower scores (i.e., more disagreement) after the synchronous stroking in the target-RUBBER condition as compared to the target-OWN condition (z = 2.24, p = 0.025; supplementary Figure S1A).

No correlations were found between the implicit (i.e., drift) and explicit (i.e., agreement with the statements) measures of the illusion (Spearman Rho < 0.28; p > 0.050).

There was a significant difference in terms of displacement_pre between the two target conditions, with bigger scores in absolute value in the target-OWN than in the target-RUBBER condition (z = −2.39, p = 0.024), indicating that subjects were less accurate in judging the location of their own hand prior to the stroking, than the location of the rubber hand.

### Experiment 2

**Figure 3:**
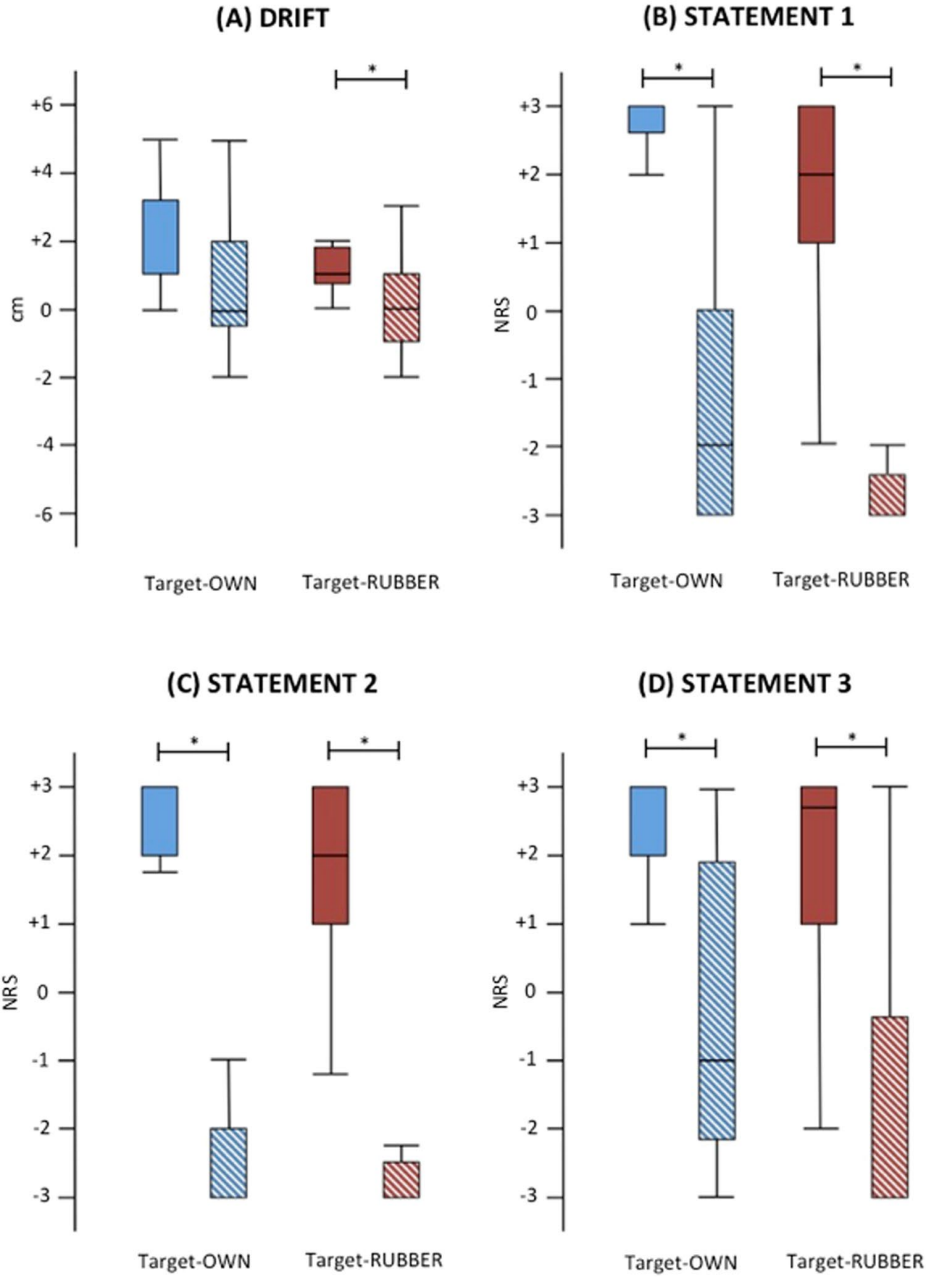
Boxplot of the drifts and statements 1 to 3 in experiment 2. The typical pattern of the rubber hand illusion in the synchronous (plain columns) compared to the asynchronous (striped columns) condition was observed for both target conditions, with the exception of the drift in the target-OWN condition. Asterisks indicate significant comparisons (p < 0.050).

The drift reached statistical significance (Friedman test, χ^2^ = 15.02, p = 0.006). This was because of significant higher scores (i.e. more agreement) after synchronous than asynchronous stroking in the target-RUBBER condition (z = 2.62, p = 0.008; Fig. 3A), whereas only a non-significant trend was detected in the target-OWN condition (z = 1.80, p = 0.067). No differences were observed between the target conditions.

The statement S1 to S3 were found to be significant (Friedman test, S1: χ^2^ = 29.83, S2: χ^2^ = 37.63, S3: χ^2^ = 28.72, all p < 0.001). For all, this was due to significant higher scores indicating more agreement after synchronous than asynchronous stroking (Fig. 3B–D), in both target-OWN (S1: z = 3.45, S2: z = 3.55, S3: z = 3.49, all p < 0.001) and target-RUBBER condition (S1: z = 3.54, p < 0.001; S2: z = 3.52, p = 0.001; and S3: z = 3.25, p < 0.001). No differences were observed between the target conditions.

With regard to the other statements, only S7 (Friedman test, χ^2^ = 12.5, p = 0.005) and S9 (Friedman test, χ^2^ = 12.43, p = 0.006) were significant. As to S7, this was due to higher scores (i.e. less disagreement) in the target-OWN condition after the synchronous stroking compared to the asynchronous stroking (z = 2.95, p = 0.003 supplementary Figure S1B). Moreover, significant higher scores were observed after synchronous stroking in the target-OWN as compared to the target-RUBBER condition (z = 2.51, p = 0.012; supplementary Figure S1B). As to S9, higher scores were observed after synchronous than asynchronous stroking in both target-OWN (z = 2.41, p = 0.016) and target-RUBBER condition (z = 2.39, p = 0.016), with no differences between the target conditions (supplementary Figure S2).

No correlations were found between the implicit (i.e., drift) and explicit (i.e., agreement with the statements) measures of the illusion (Spearman Rho < 0.26; p > 0.050).

There was no difference in terms of displacement_pre between the two target conditions (Friedman test, χ^2^ = 0.43, p > 0.05), indicating that localization accuracy before stroking was similar for both hands.

### Experiment 1 vs experiment 2

**Figure 4:**
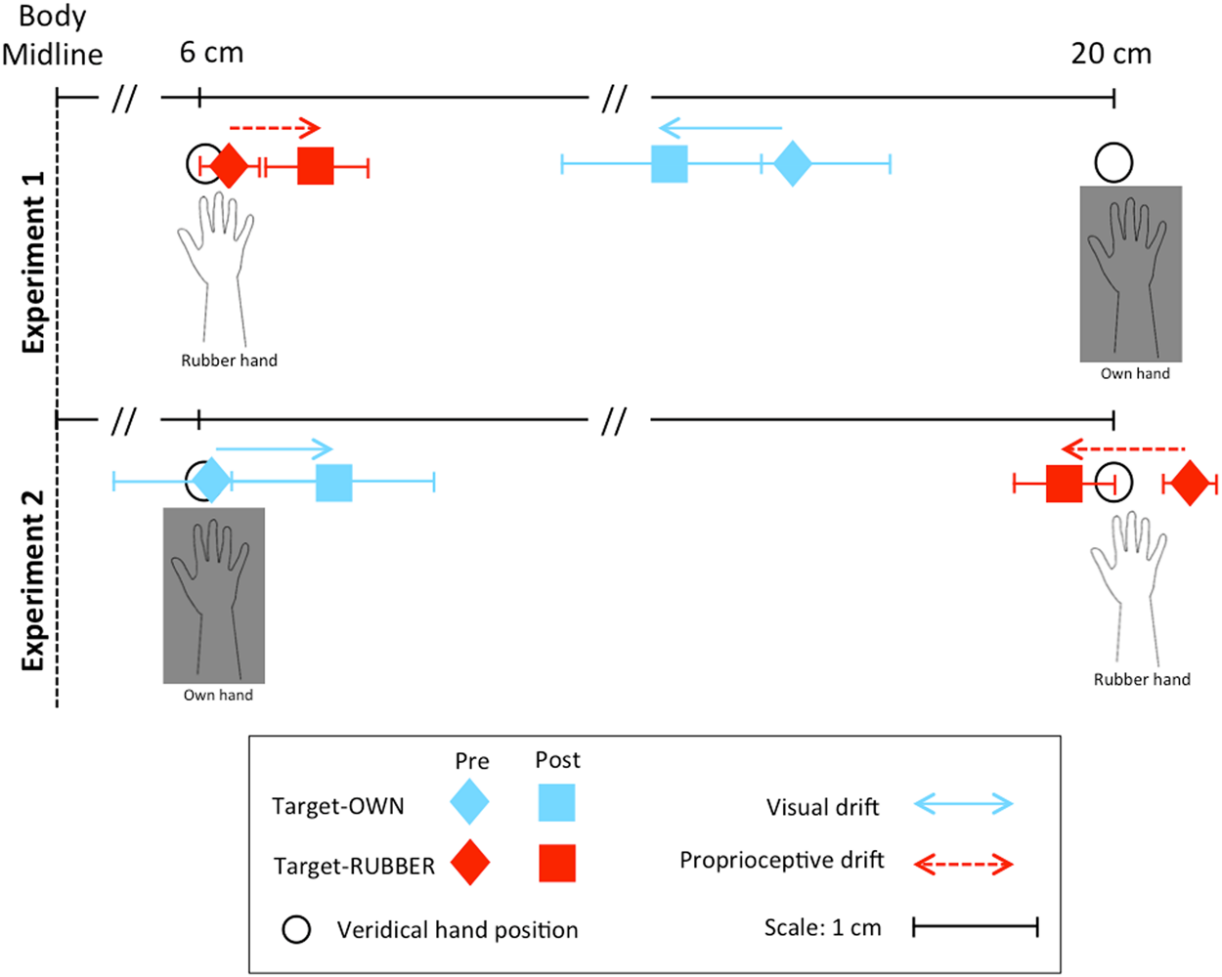
Localization judgments before and after the synchronous stroking in both target conditions. The accuracy of the (proprioceptive) localization of the own hand before the stroking increases when it is placed near the midline, while this does not hold true for the (visual) localization of the rubber hand. Plain arrows indicate the visual drifts going in the opposite direction to dashed arrows that indicate the proprioceptive drifts.

There were no differences between the two experiments, with only two exceptions: 1) the drift after the asynchronous stroking in the target-OWN condition was significantly higher in experiment 2 than in experiment 1 (z = −2.39, p = 0.024), indicative of a large proprioceptive drift after the asynchronous stroking when the participants’ own hand was close to the midline; and 2) the displacement_pre in the target-OWN condition was significantly bigger in absolute value in experiment 1 than in experiment 2 (z = −2.30, p = 0.021), indicative of higher accuracy in the localization of the own hand when this is close to the midline. Figure 4 summarizes the displacement errors before and after synchronous stroking in both target conditions and in both experiments: It further demonstrates how, in each experiment, the proprioceptive drift is mirrored by a *visual* drift in the opposite direction, which argues for a convergence of the percepts of two hands towards each other.

## Discussion

The main finding of the current work is that the RHI induces a *visual drift* (i.e., a perceptual mislocalization) of the rubber hand towards the subject’s hidden hand after synchronous stroking that is in the opposite direction to the most known proprioceptive drift. This confirms and expands on the results of Fuchs *et al*. that, as a result of the illusion, the spatial representations of the rubber and own hands converge to each other^21^.

In the canonical “target-OWN” condition, we largely replicated findings from previous studies using the RHI paradigm^3,4,13,23,24^, both in terms of the proprioceptive drift and of the subjective feelings related to the illusion. This was the case in both experiments with the exception of the proprioceptive drift in experiment 2 that did not reach the significance threshold, owing to a large drift after the asynchronous stroking. Hence, we support previous proposals that the asynchronous stroking might induce a rudimentary form of the illusion^21,25,26^. This finding occurred only in experiment 2 (i.e., when the participants’ own hand was placed medial to the rubber hand). Whether this spatial configuration could induce an easier tendency to recalibrate the hand remains open to discussion.

The main result of the current work, however, is that a similar and complementary displacement (i.e., the visual drift) could be observed in the “target-RUBBER” condition (Fig. 4). Importantly and differently from Fuchs *et al*.^21^, the magnitude of the displacement after the synchronous stroking was bigger (in absolute value) than the perceived displacement before the stroking, indicating an *actual* approach of either hand towards the other. The latter finding might be due to the longer time of the stroking in our study (2 minutes) compared to Fuchs *et al*. (30 to 60 sec)^21^. In fact, there is evidence that the proprioceptive drift might increase as a function of the time of stroking^4,27^. Moreover, in their study localization judgments after stroking were made in comparison to a “no-hand” condition, which served as a proxy of localization accuracy based on the visual modality, rather than to a pre-stroking measure^21^. Across the stroking, subjects had to localize two different objects within the same visual field, each with its own size and shape (i.e., a small dot vs the tip of the rubber hand index) and this might have affected the results^28^. Finally, they asked their subjects to perform a pointing movement with the contralateral arm for localizing either hand^21^, while we collected a visual measure. Besides the differences discussed above, there is further evidence that the two approaches are differently sensitive to the RHI, with pointing movements being more implicit than visual measures and more strongly tied to a proprioceptive and action-related space^29^. This is consistent with the model of multiple body representations, which separates the body schema for motor responses (that is less prone to the illusion) from the body image underlying pure perceptual judgments (that is sensitive to the illusion)^29^.

The evidence that the spatial representations of the own and rubber hands converge towards each other would be in keeping with principles of multisensory integration according to which the brain predicts a weighted sum of single-cue estimates^5,11,12,17–20,30^. The reciprocal attraction of the two hands might suggest that conflicting sensory information are integrated into a common percept that would be placed in between the two hands. Such integration of conflicting sensory information has been also demonstrated for vision and touch^31^: localization judgments of a body part do not involve just a simple dominance of vision over other sensory modalities (in our case proprioception) but a genuine multisensory interaction.

However, just as in Fuchs *et al*.^21^, the spatial representations of the two hands did not overlap completely (i.e., the drifts did not fully converge). This might be explained by the fact that, despite the illusion, subjects are well aware that the rubber hand is placed at a certain distance from their own hand: it is therefore conceivable that top-down constraints prevent subjects to directly point to an intermediate percept. We predict that an increased overlap between the spatial representations of the two hands can be obtained by reducing the distance between the two hands^32^.

As to the subjective feeling of the illusion, we largely replicated previous findings that the sense of embodiment mostly arises after the synchronous stroking^3,4,13,23,24^. This occurred in both target conditions, indicating that the focus on either hand does not greatly influence the subjective feeling of the illusion, and in both experiments, suggesting that the relative position of the two hands (one lateral and one medial) also does not influence the illusion.

One interesting result was observed with regard to S7 (i.e., “It felt as if my hand were turning rubbery”). In both experiments we observed a consistent pattern with subjects indicating uncertainty in the target-OWN condition and frank disagreement in the target-RUBBER condition (supplemental Figure S1). This consistently reached significance in both experiments, suggesting that it is not a spurious finding. It is not clear which subjective aspect of the RHI is depicted by S7^24^ and if it refers more to the embodiment of the rubber hand or to the “loss of own hand”. Our results (i.e., S7 not paralleling the sense of ownership over the rubber hand as depicted by S3) might preliminary support the latter. If so, different S7 scores along with similar S3 scores in the two target conditions might imply that the embodiment of the rubber hand might occur either as a substitution of the participant’s body part that is hidden from view^3,4,13^ or in addition (i.e., ownership over a supernumerary hand)^27,33–35^. It is not straightforward how this occurred. However we note that, despite the experimental procedure being exactly the same for both target conditions, participants were exposed to the task of localizing the rubber hand prior to the stroking in the target-RUBBER condition. This might have “increased” the focus on the rubber hand. In other words, participants might have paid more attention to the rubber hand based on the expectation to localize it at the end of the stroking (and vice-versa in the canonical target-OWN condition). This could have facilitated the different responses as to S7 (i.e., “It felt as if my hand were turning rubbery”). Further research is warranted to fully explain this finding.

A difference between synchronous and asynchronous conditions emerged also in another “control” statement, (i.e., S9: “The rubber hand began to resemble my own hand, in terms of shape, skin tone, freckles or some other visual feature”), which reached significance in experiment 2 (supplementary Figure S2). This could be interpreted as a further aspect of the illusion (i.e., similarity between the two hands), as suggested in a previous study^24^.

As a corollary finding, we further demonstrated that the displacement error before stroking (thus, unrelated to the illusion) in the “target-OWN” condition was larger in experiment 1 than experiment 2 (Fig. 4). This is consistent with the evidence that proprioceptive judgments lose accuracy (and are most susceptible to the midline bias) when the lateral distance between the arm and the trunk (i.e., midline) is increased^12,22^. This was the reason thereby we designed our experiment 2 in which the position of the two hands was interchanged.

We acknowledge that, as stated in the methods, the “target-RUBBER” condition was performed always after the “target-OWN” condition, raising the potential bias of a carryover effect. However, in a previous study in which the RHI was repeated for 3 consecutive days no significant across-sessions effect was observed^36^. Other studies further suggested that the opposite effect (i.e., dissipation of the illusion rather than a carryover effect) might occur with repeated trials within a single session of RHI^21,37^. We do not believe this was the case in our study since no differences were detectable in both experiments between the target conditions in terms of the strength of the illusion. It might be that testing each subject at least 4 weeks apart has reduced this potential bias. Moreover, while we cannot entirely exclude a bias as to the subjective reports of the illusion (either in terms of carryover effect or habituation), we also note that our participants were never exposed to the task of localizing the rubber hand when attending the second experiment and, as such, the “visual” drift should have not been majorly influenced.

With the rubber hand being out of view during the judgments, it might be argued that the localized percept is not strictly based on vision, but rather on a visual memory or afterimage^38^. However, from a methodological point of view this should not have affected our results since it has been demonstrated that the visual representation of an object near the body almost coincides with its spatial representation when vision is occluded^39^. This is reflected in our experiments by the very high accuracy in localizing the rubber hand (again when it was out of view) prior to the stroking.

In conclusion, we support the suggestion raised by Fuchs *et al*.^21^ that there is an actual convergence of the spatial representations of the two hands. This finding would challenge common interpretations of the RHI whereby the proprioceptive drift reflects a substitution of the subjects’ own hand by the rubber hand.

## Electronic supplementary material

Supplemental figures
