# Supplementary material for: Judging the position of the artificial hand induces a “visual” drift towards the real one during the rubber hand illusion
Source: Sci Rep. 2018 Feb 7;8:2531. doi: 10.1038/s41598-018-20551-6 (PMC5803228; doi:10.1038/s41598-018-20551-6)

Judging the position of the artificial hand induces a "visual" drift towards the real one during the rubber hand illusion

Roberto Erro[1,2], Angela Marotta[1,3], Michele Tinazzi[1], Elena Frera[1], Mirta Fiorio[1]

1. Department of Neurosciences, Biomedicine and Movement Sciences, University of Verona, Verona, Italy
2. Center for Neurodegenerative Diseases (CEMAND). Department of Medicine, Surgery and Dentistry "Scuola Medica Salernitana", University of Salerno, Baronissi (SA), Italy
3. Neurology Unit, Neuroscience Department, AOUI Verona, Italy

Figure S1. Statement 7. Boxplots depicting the scores for statement 7 in experiment 1 (A) and experiment 2 (B) showing a similar pattern. Namely, subjects gave lower scores, indicating more disagreement, after the synchronous stroking in the target-RUBBER condition as compared to the target-OWN condition (A and B). Asterisks indicate significant comparisons (p<0.050).

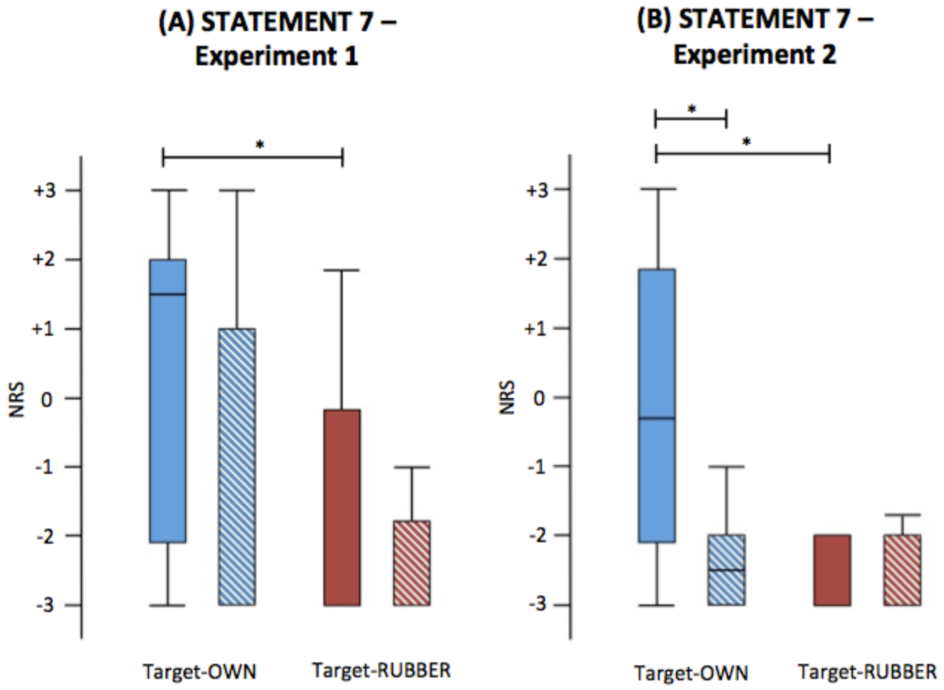

Figure S2. Statement 9. Boxplot depicting scores for statement 9 in experiment 2 showing the typical pattern of the rubber hand illusion, with higher scores in the synchronous compared to the asynchronous condition, and confirming "similarity" as a further aspect of the illusion. Asterisks indicate significant comparisons ($p<0.050$).

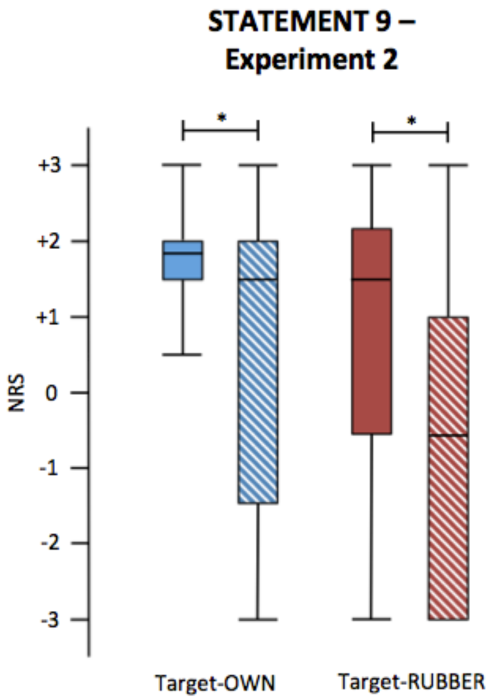

Supplement: Supplementary file 1 — Supplemental figures [file 41598_2018_20551_MOESM1_ESM.pdf]
